# Creosote

**Published:** 1836

**Authors:** 


### Art. III.—Creosote.

Our readers are aware that this new medicinal substance, the product of repeated distillations of the oil which comes over with the tar in the process of making pyroligneous acid, has been employed in various affections. M. Reichenbach discovered it, and called it "Kreosote—from *kreos*—flesh, and *sozo*—I save, and like every discoverer of a new medicine, he has lavished great praise upon its therapeutical powers. He says, that in his hands, it proved a cure for cairies, cancer, carcinomatous ulcers, rheumatism, pneumonic diseases, &c.

Dr. Elliotson, of the London University, has lately, as we learn from the April number of Johnson's Medico-Chirurgical Review, published a paper eulogistic of Creosote, as effectually remedial in several severe varieties of morbid affection.

1. Phthisis—internally given, Creosote was of no avail, but employed in the way of inhalation, by dissolving it in mucilage and water, and making the patient breathe through the mixture, it mitigated the cough and expectoration, and increased the facility of respiration.

2. Neuralgia—given in doses of from one to seven drops, three times a day, it proved very beneficial in this disease.

3. Vomiting—the power of the remedy in arrresting vomiting where no gastritis exists, is perfectly established according to the testimony of both Drs. Elliotson, and Roots.

Dr. Elliotson says, "I have repeatedly seen it succeed after the failure of Prussic acid, which is the most powerful remedy I previously was acquainted with in subduing the action of the stomach." He advises it in doses of from two to three drops every one, two, or three hours, as the case may be. In cholic it will arrest vomiting, and thus secure the action of the other medicines by enabling the stomach to retain any cathartic which it may be thought proper to administer. In vomiting from pregnancy it is very available in quieting the stomach.

4. Dyspepsia—in some forms of this affection, where no inflammation or structural disease of the abdominal viscera exists, it is a superior remedy.

5. Chronic Glanders—affecting one nostril and the frontal sinuses, contracted from a glandered horse—was subjected to a "sedulous injection of a weak solution of creosote up the nostril," and the "whole of the symptoms, after a very few weeks," were removed.

In Epilepsy, Diabetes, and achne indurata—the remedy seemed to have proved ineffectual.

Dr. Roots regards creosote as holding the same rank in the Materia Medica, as a remedy in irritable conditions of the stomach, free from all inflammatory action, as does the Oxide of Bismuth."

